# Transcriptional Response of Blood Mononuclear Cells from Patients with Inflammatory and Autoimmune Disorders Exposed to “Krakow Smog”

**DOI:** 10.3390/cells11162586

**Published:** 2022-08-19

**Authors:** Adrianna Gałuszka-Bulaga, Jacek Hajto, Małgorzata Borczyk, Sławomir Gołda, Marcin Piechota, Michał Korostyński, Magdalena Rutkowska-Zapała, Paweł Latacz, Zofia Guła, Mariusz Korkosz, Joanna Pera, Agnieszka Słowik, Maciej Siedlar, Jarek Baran

**Affiliations:** 1 Department of Clinical Immunology, Institute of Pediatrics, Jagiellonian University Medical College, 30-663 Krakow, Poland; 2 Laboratory of Pharmacogenomics, Department of Molecular Neuropharmacology, Maj Institute of Pharmacology, Polish Academy of Sciences, 31-343 Krakow, Poland; 3 Department of Clinical Neurology, Jagiellonian University Medical College, 30-688 Krakow, Poland; 4 Department of Rheumatology and Immunology, Jagiellonian University Medical College, 30-688 Krakow, Poland

**Keywords:** air pollution, gene expression, inflammatory, autoimmune disorders

## Abstract

Despite the general awareness of the need to reduce air pollution, the efforts were undertaken in Poland to eliminate the pollutants and their harmful effect on human health seem to be insufficient. Moreover, the latest data indicate that the city of Krakow is at the forefront of the most polluted cities worldwide. Hence, in this report, we investigated the impact of particulate matter isolated from the air of Krakow (PM KRK) on the gene expression profile of peripheral blood mononuclear cells (PBMCs) in healthy donors (HD) and patients with atherosclerosis (AS), rheumatoid arthritis (RA) and multiple sclerosis (MS), after in vitro exposure. Blood samples were collected in two seasons, differing in the concentration of PM in the air (below or above a daily limit of 50 µg/m^3^ for PM 10). Data show that PBMCs exposed in vitro to PM KRK upregulated the expression of genes involved, among others, in pro-inflammatory response, cell motility, and regulation of cell metabolism. The transcriptional effects were observed predominantly in the group of patients with AS and MS. The observed changes seem to be dependent on the seasonal concentration of PM in the air of Krakow and may suggest their important role in the progression of AS, MS, and RA in the residents of Krakow.

## 1. Introduction

Although the awareness of the harmful effects of air pollution on human health is growing rapidly, there is still no clarity on how particulate matter (PM) affects the human gene expression profile. This is of relevance as, alongside genetic predispositions and epigenetic factors, environmental pollution plays an important role in the initiation and development of many pathologies [1,2,3]. For example, it is commonly accepted that long-lasting exposure to air pollution is associated with an increased risk of cardiopulmonary disorders, morbidity, and mortality [4,5,6]. Currently, Poland holds the infamous leading position among the countries where the concentration of PM in air frequently exceeds a daily limit of 50 µg/m^3^ for PM 10 and 25 µg/m^3^ for PM 2.5 [7,8,9,10]. Despite the local regulations reducing the air pollution in the most polluted cities, the concentration of PM in the air during a year is still above the limits established by the World Health Organization (WHO) [11,12]. This is observed mostly in southern Poland. Krakow is one such city, with the PM limits being significantly exceeded, especially in the wintertime [11]. According to the IQAir air quality ranking, on 14 December 2021, Krakow was the most polluted city in the world. On this day, the concentrations of PM 10 and PM 2.5 in the air exceeded by a fold of four the accepted levels and reached 230 µg/m^3^, and more than 100 µg/m^3^, respectively [13]. The first place of Krakow in the ranking list of the most polluted cities may be surprising, as in 2019 the city council adopted and implemented the anti-smog resolution. However, the main reason that Krakow cannot cope with such a high air pollution is its location, which limits the movement of the air masses above the city. Additionally, unfavorable neighborhoods, such as steel mills, power plants, chemical factories, and the combustion of solid fuels for house heating potentiates this problem [14]. Air pollution increases the social burden and leads to the deterioration of people’s life quality [15,16]. PM 10, and especially PM 2.5, are considered the most harmful to human health [15,17,18,19,20,21,22]. They can penetrate the lower respiratory tract and translocate from the lung to the bloodstream, which may affect the functions of blood erythrocytes, platelets, and leukocytes [23,24,25,26]. Moreover, it was shown that ambient PM might contribute to the initiation and development, among others, of atherosclerosis (AS) [27], rheumatoid arthritis (RA) [3], and multiple sclerosis (MS) [28]. Data from our previous reports suggests the role of air pollution in the stimulation of the pro-inflammatory response of Th1 and Th17 cells [29]. This effect was observed both in healthy donors (HD) and patients with rheumatoid arthritis (RA), multiple sclerosis (MS), and atherosclerosis (AS), and was strongly dependent on monocytes and the seasonal variations in the concentration of PM in the air of Krakow (PM KRK) [30]. So far, the impact of PM on immune cell functions has been well described [29,30,31,32]. Moreover, the list of evidence showing that air pollution affects gene expression has been continuously growing [33,34,35,36]. A recent study revealed that short-term exposure to PM induces compound-specific expression of blood cell genes and microRNAs profile [37]. In another study, Croft et al. also showed that air pollution is associated with changes in gene expression within innate immunity-related pathways [38]. Studying the gene expression changes may therefore provide a better understanding of the regulatory mechanisms in response to ambient air pollutants.

Considering our previous results and new evidence, here we aimed at investigating the transcriptional effects of PM KRK, including possible seasonal variation, on the gene expression profile of peripheral blood mononuclear cells from HD and patients with AS, RA, and MS.

## 2. Materials and Methods

### 2.1. Patients and Control Groups

Overall, 36 patients were enrolled in the study between 14 June 2019 and 15 February 2021. The group contained 9 patients with RA—recruited in the Department of Rheumatology and Immunology, Jagiellonian University Medical College in Krakow, and classified with new-onset RA before introducing treatment with glucocorticosteroids (GC) and/or Disease-Modifying Anti-Rheumatic Drugs (DMARD); 9 patients with MS and 9 patients with AS—diagnosed at the Department of Clinical Neurology, Jagiellonian University Medical College in Krakow, based on the McDonald criteria for MS [39] and TOAST criteria for AS [40], respectively. Patients’ blood (10 mL) was drawn into EDTA-containing Vacutainer tubes (BD Vacutainer, San Jose, CA, USA) and processed within 2 h. In parallel, blood samples from 9 healthy donors (HD) were commercially obtained from the Regional Center of Blood Donation and Blood Therapy in Krakow, Poland, and used as controls. All the procedures involving patients were approved by the local Jagiellonian University Bioethical Committee (approval no. 122.6120.261.2015). Basic characterization of the patients and healthy donors (mean age, sex ratio) and the frequencies of the disease’s prevalence in the local population were already documented [30]. Briefly, data showed that the mean age of patients was 30.88 ± 7.36 for MS, 43.55 ± 10.51 for RA, 69.90 ± 11.02 for AS, and 38.90 ± 11.17 for HD. The reported sex ratio (female to male) was 1.670 for MS, 1.750 for RA, 0.538 for AS, and 0.538 for HD, respectively.

### 2.2. Preparation of the PM from the Air of Krakow

Particulate matter from the air of Krakow (PM KRK) was collected between 2018 and 2019 in the urban area (city center) of Krakow (marked as Urban B), by a custom-designed system, using 16 polytetrafluoroethylenes (PTFE) filters (diam. 47 mm, pore size 2.2 μm), as described previously [41]. The system did not possess a size separation unit, so particles of different sizes were collected simultaneously. Filters were changed every week and air pollutants were extracted from the filters, dried, and pooled in the Department of Inorganic Chemistry, Faculty of Chemistry, Jagiellonian University in Krakow, Poland, as described previously [41]. A general physicochemical analysis of the collected PM, covering carbon, hydrogen, nitrogen, and sulfur content was performed by our partners from the Faculty of Chemistry, Jagiellonian University [41]. Preparations of PM were weighted on a high precision microbalance and suspended in RPMI 1640 medium (Corning, Manassas, VA, USA) under sterile conditions. The final concentration of PM KRK (10 µg/mL) was established experimentally as non-toxic for PBMCs. Cell viability was assessed after exposure to PM KRK by flow cytometry, using Annexin V Apoptosis Detection Kit I (BD Pharmingen, San Diego, CA, USA) according to the manufacturer’s instructions. Briefly, PBMCs after 3 h of culture with or without PM KRK were harvested, washed in PBS (Corning), resuspended in binding buffer, stained with Annexin V-FITC and propidium iodide (PI) (15 min at room temperature), and examined by flow cytometry (FACSCalibur, BD Biosciences Immunocytometry Systems, San Jose, CA, USA). Typically, 10,000 events were acquired for analysis [30]. On the day of blood collection, the concentration of PM 10 in the air of Krakow, in the summer and winter periods in 2019–2021, was recorded [30]. Briefly, mean concentration of PM 10 and PM 2.5 (µg/m^3^ ± SD) in the air of Krakow was reported as 17.53 ± 9.27 and 9.98 ± 5.46 in the summer and 76.43 ± 36.77 and 50.46 ± 27.02 wintertimes, respectively.

### 2.3. Cell Isolation

Blood samples were collected in two different seasons when the concentration of PM 10 in the air of Krakow was lower than the daily limit of 50 µg/m^3^ (summer), and when it was higher than 50 µg/m^3^ (winter). Peripheral blood mononuclear cells (PBMCs) were isolated by standard Pancoll (Panbiotech, Aidenbach, Germany) density gradient centrifugation, washed and resuspended in RPMI 1640 medium (Corning, Manassas, VA, USA), supplemented with 2 mM of L-glutamine, 5% heat-inactivated fetal bovine serum (EURx, Gdańsk, Poland), and 25 µg/mL gentamycin (Sigma, St. Louis, MO, USA) (complete medium). 

### 2.4. Cell Culture

PBMCs were cultured at the density of 1 × 10^6^/mL in ultra-low-attachment tubes (Corning, Manassas, VA, USA) in complete RPMI 1640 medium, with or without the addition of PM KRK, used at the concentration of 10 µg/mL. Cells were kept at 37 °C, 5% CO_2_ in a humidified atmosphere. As a positive control, cells were stimulated with 50 ng/mL PMA (phorbol 12-myristate 13-acetate; Sigma) and 100 ng/mL of Ionomycin (Sigma). After 3 h of culture, the cells were harvested, and washed once in PBS (phosphate-buffered saline; Corning, Manassas, VA, USA) with 5% heat-inactivated fetal bovine serum (EURx).

### 2.5. RNA Isolation

RNA was isolated following the manufacturer’s protocol and further purified using the RNeasy Mini Kit (Qiagen, Hilden, Germany). The total RNA concentration was measured using an ND-1000 Spectrometer (NanoDrop Technologies Inc., Wilmington, DE, USA). The quality of RNA was determined by using an RNA 6000 Nano Lab-Chip Kit and an Agilent Bioanalyzer 2100 (Agilent Technologies, Palo Alto, CA, USA). Based on the RNA integrity number (RIN > 7.5) values, 36 samples were selected for sequencing. 

### 2.6. Library Preparation for mRNA Sequencing

A total amount of 1 μg RNA per sample was used as input material for the RNA sample preparations. mRNA from eukaryotic organisms was enriched using oligo(dT) beads from NEBNext Poly(A) mRNA Magnetic Isolation Module (cat. no. E7490L-NEB, Ipswich, MA, USA). Subsequently, sequencing libraries were generated using NEBNext Ultra II Directional RNA Library Prep Kit for Illumina (cat. no. E7770L-NEB) following the manufacturer’s recommendations. Briefly, fragmentation was carried out using divalent cations under elevated temperature in NEBNext First Strand Synthesis Reaction Buffer (5X). First-strand cDNA was synthesized using random hexamer primer and M-MuLV Reverse Transcriptase (RNase H-). Second strand cDNA synthesis was subsequently performed using DNA Polymerase I and RNase H. In the reaction buffer, dNTPs with dTTP were replaced by dUTP. The remaining overhangs were converted into blunt ends via exonuclease/polymerase activities. After adenylation of 3′ ends of DNA fragments, NEBNext adaptors with hairpin loop structure were ligated to prepare for hybridization. To select cDNA fragments of preferentially 250~300 bp in length, the library fragments were purified with AMPure XP beads (cat. no. A63987; Beckman Coulter, Beverly, MA, USA). Then, 3 μL USER Enzyme (NEB) was used with size-selected, adaptor-ligated cDNA at 37 °C for 15 min. followed by 5 min. at 95 °C before PCR. Then PCR was performed with Phusion High-Fidelity DNA polymerase, Universal PCR primers, and Index (X) Primer. At last, products were purified (AMPure XP beads) and library quality was assessed using the Agilent High Sensitivity DNA Kit (cat. no. 5067-4626) on the Agilent Bioanalyzer 2100 system (Agilent Technologies).

### 2.7. Clustering and Sequencing

Sequencing was performed by the Novogene Experimental Department (Tianjin, China). The clustering of the index-coded samples was performed on a cBot Cluster Generation System (cat. no. SY401-2015; Illumina) using TruSeq PE Cluster Kit v3-cBot-HS (cat. no. PE-401-3001; Illumia) according to the manufacturer’s instructions. After cluster generation, the libraries were sequenced on a NovaSeq 6000 Illumina platform using NovaSeq 6000 S2 Reagent Kit v1.5 (cat. no. 20028314—(300 cycles)) and 150 bp paired-end reads were generated (minimum 12 Gb and 40 M per sample).

### 2.8. RNA-Seq Analysis

All samples were checked for quality with fast QC v. 11.8 and aligned to the human reference genome (grch38 from index provided by hisat2) with hisat2 2.1.0. Cufflinks v. 2.2.1 package and GTF from the Ensemble gene database (release 104) were used to quantify (cuff-quant) and normalize (cuff-norm) transcripts to FPKMs (Fragments Per Kilobase of transcript per Million fragments mapped).

### 2.9. Statistics

Statistics for the gene expression analysis were performed using R software. Genes with median FPKM <1 were excluded from the analysis. Statistical significance was tested after quantile normalization using repeated ANOVA measures (stimulation × group) on log_2_ (1 + FPKM) values with a false discovery rate adjustment. For post-hoc analysis, pairwise *t*-tests with Bonferroni corrections were performed for all genes that passed the 1% FDR (False Discovery Rate) threshold for the following given factor: stimulation (paired *t*-tests) or group (unpaired *t*-tests). Fold change was calculated as a difference of mean FPKM values between relevant groups (log2 ratio). For analysis of seasonal variation three-way ANOVA (stimulation × group × season) was used with stimulation as a repeated measure and without the three-factor interaction. Gene functional enrichment analysis was performed with gene annotation online tool Enrichr (ref: https://currentprotocols.onlinelibrary.wiley.com/doi/10.1002/cpz1.90; accessed on 30 May 2022) [42,43,44]. The overrepresentation of regulated genes in the GO:1900017 term was assessed using Fisher’s exact test.

## 3. Results

### 3.1. Disease Type and Stimulation with PM KRK Affect the Gene Expression Profile in PBMCs

**Figure 1:**
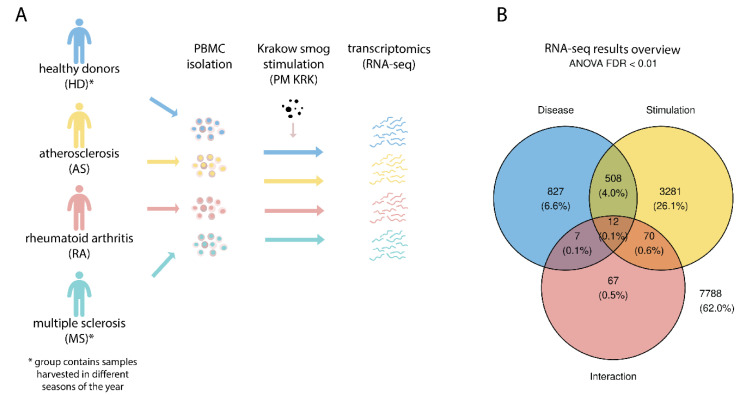
Overview of the study. (**A**) Peripheral blood mononuclear cells (PBMCs) were isolated from healthy donors (HD) and patients with rheumatoid arthritis (RA), multiple sclerosis (MS), and atherosclerosis (AS). Cells were then stimulated with smog particles (PM KRK) and their transcriptome analyzed and compared to non-stimulated cells. (**B**) Transcript counts were transformed, normalized, and analyzed with two-way ANOVA. The Venn’s diagram shows each factor’s number of differentially expressed genes.

The effect of local air pollution on the gene expression profile in PBMCs was evaluated after a 3-h exposure of patients and HD PBMCs to PM KRK. Cells were obtained from peripheral blood and after their exposure to PM KRK, the total RNA was isolated, and the transcriptome of cells was analyzed (Figure 1A). Two RNA samples (one from RA and one from the MS group) were excluded from the further analysis based on the low concentration or low quality of RNA. The Venn’s diagram revealed that 13.9% of 4772 differentially expressed genes were regulated both by the disease and stimulation factors, as 664 genes showed either interaction or both, the disease and stimulation factor below the threshold (Figure 1B).

Transformed FPKM values for the analyzed genes, results of ANOVA, and post-hoc analyses have been presented in Table S1. Overall, 1354 genes passed the FDR corrected *p* < 0.01 for the disease factor (AS, RA, MS), while 3871 genes were regulated by the stimulation factor (PM KRK), and 156 by interaction. For a heatmap presentation, genes with the most significant *p*-value were shown [42,43,44]. Results for each of the factors are described in subsequent sections.

### 3.2. Effect of Disease on the PBMCs Gene Expression Profiles

**Figure 2:**
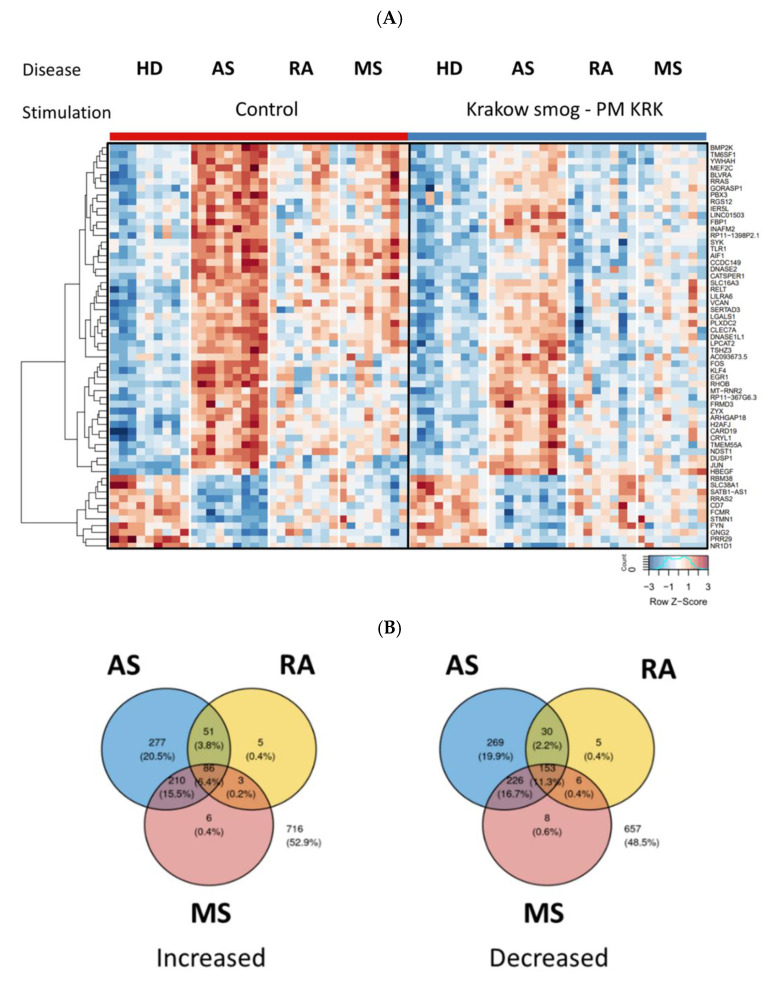
Gene expression differences between the PBMCs derived from healthy donors (HD) and patients with rheumatoid arthritis (RA), multiple sclerosis (MS), and atherosclerosis (AS). (**A**) RNAseq results are shown as a heatmap and include transcripts with a genome-wide significance from two-way ANOVA for the disease factor (FDR corrected *p* < 0.001) and the difference between control vs. disease group (a fold of change log_2_ > 1). The intensity of the coloured rectangles represents transcript abundance levels. The presented level is proportional to the row z-score values (between −3 and 3) as displayed on the bar below the heatmap image. Hierarchical clustering was performed using correlation as a distance measure. The full list of differentially expressed transcripts is presented in Table S1. (**B**) Comparison of the number of genes with expression altered by diseases. The results of ANOVA analysis for disease factor (threshold *p* < 0.01 FDR corrected) followed by post hoc test (*p* < 0.01) between healthy and disease groups.

When we compared the gene expression level by disease factor without PM KRK stimulation, significant differences between the groups of patients (AS, RA, and MS) and HD were detected (Figure 2A). To verify the effect of the disease, we compared the gene expression profiles in the patients’ groups and HD (as a reference) regardless of whether the cells were stimulated with PM KRK or not. In this aspect, the expression of most genes affected by disease factors was distinct in AS when compared to HD (post-hoc analysis, 1302 genes with *p*-value < 0.01, including 624 upregulated and 678 downregulated) (Figure 2B). In the cases of MS and RA, the number of genes with affected expression compared to HD was 698 (305 upregulated; 393 downregulated) and 339 (145 upregulated, 194 downregulated), respectively (Figure 2B). Gene-set enrichment analysis with the BioPlanet database revealed multiple pathways enriched in the genes regulated by the studied diseases (Table S2). These include various biological processes such as BDNF, IL-3, IL-1, IL-5, MAPK, and integrin dependent signaling pathways.

### 3.3. The PM KRK Treatment Effects on PBMCs Gene Transcription Profiles

**Figure 3:**
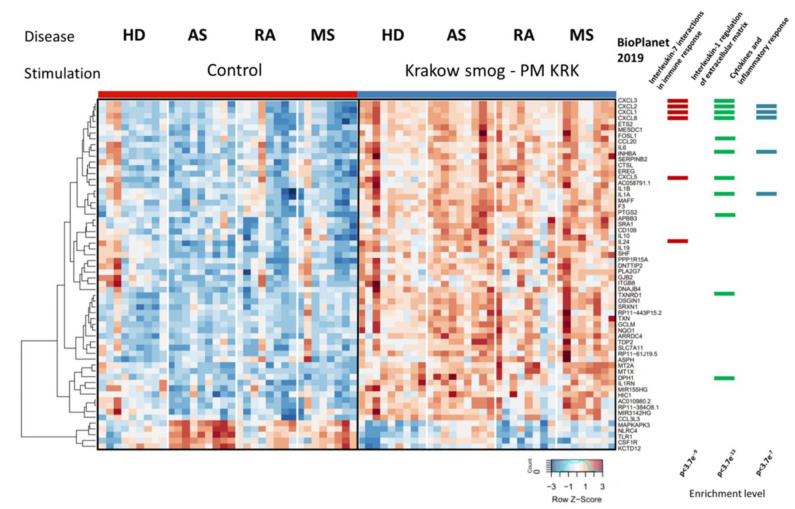
Gene expression alterations induced by PM KRK stimulation. The PBMCs-derived from healthy donors (HD), patients with atherosclerosis (AS), rheumatoid arthritis (RA), and multiple sclerosis (MS) were treated with PM KRK. RNAseq results are shown as a heatmap and include transcripts with a genome-wide significance from two-way ANOVA for the stimulation factor (FDR corrected *p* < 10^−9^) and the difference between the control and stimulation group (a fold of change log_2_ > 1). The intensity of the coloured rectangles represents transcript abundance levels. The presented level is proportional to the row z-score values (between −3 and 3) as displayed on the bar below the heatmap image. Hierarchical clustering was performed using correlation as a distance measure. The full list of differentially expressed transcripts is presented in Table S1. On the right panel, the results of the functional enrichment analyses performed with the Enrichr tool are presented. Three example over-represented pathways are based on BioPlanet 2019 dataset. The full list of enriched biological terms is presented in Table S2.

When we compared the gene expression level with respect to the stimulation factor (unstimulated cells vs. stimulated with PM KRK), we observed differences in all groups (HD, AS, RA, and MS) (Figure 3). Stimulation with PM KRK affected the expression of 513 genes in the control HD group (including 248 upregulated and 265 downregulated), 1678 genes in AS (729 upregulated, 949 downregulated), 249 genes in MS (91 upregulated, 158 downregulated), and 191 genes in RA (111 upregulated, 80 downregulated). Genes affected by PM KRK stimulation were enriched in genes involved mostly in immune and inflammatory responses, IL-1, IL-2, IL-4, IL-5, IL-7, IL-17, and Toll-like signaling pathways (e.g., IL1A, IL6, CXCL1, CXCL2, and CXCL8) (Table S2). 

### 3.4. Disease-Dependent Differences in the Transcriptional Response of PBMCs to PM KRK Treatment

**Figure 4:**
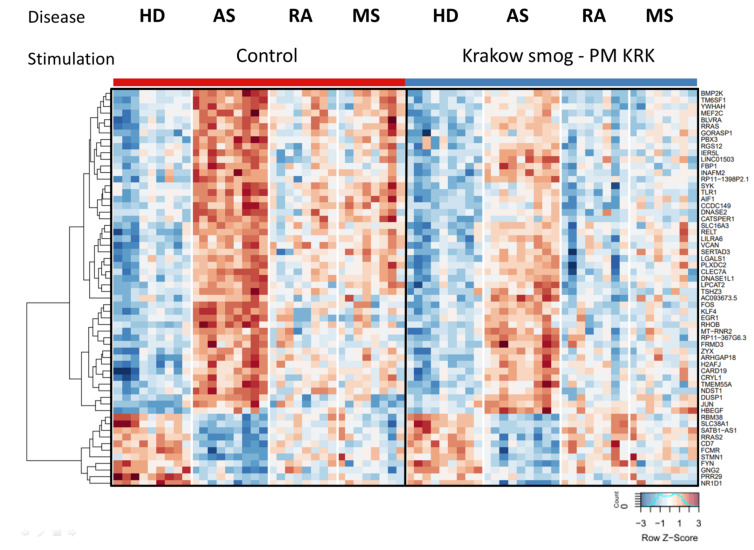
Gene expression differences between the response of PBMCs derived from HD, RA, MS, and AS to PM KRK treatment. RNAseq results are shown as a heatmap and include transcripts with a genome-wide significance from two-way ANOVA for the interaction (FDR corrected *p* < 0.01) and the difference between disease or stimulation group vs. the appropriate control (a fold of change log_2_ > 1). The intensity of the coloured rectangles represents transcript abundance levels. The presented level is proportional to the row z-score values (between −3 and 3) as displayed on the bar below the heatmap image. Hierarchical clustering was performed using correlation as a distance measure. The full list of differentially expressed transcripts is presented in Table S1.

Next, in all studied groups, the differential gene expression profiles were analyzed for the interaction of the factors (disease and stimulation). The most pronounced effect of the interaction was detected for 156 genes, of which 60 were presented on the heatmap (Figure 4). To determine the biological pathways related to the genes listed in Figure 4, the enrichment analysis was performed using the BioPlanet resource. The results of this analysis are presented in Table 1.

### 3.5. PM KRK Treatment Upregulates the Expression of Genes Responsible for Pro-Inflammatory Cytokine Production

We selected genes related to the regulation of cytokine production as our previous results [30] indicated that air pollution induces a complex pro-inflammatory response. The biological term GO:1900017 was used to prepare a comprehensive list of genes involved in the positive regulation of inflammatory response. The set consisted of 23 distinct genes, of which 18 were expressed in PBMCs (median FPKM > 1). In total, 12 of them were significantly differentially expressed after stimulation (FDR *p* < 0.01), and Fisher’s exact test showed an over-representation of significantly regulated genes at *p* < 10^−15^ as compared with randomly drawn gene sets of the same length (Table 2). The most expressed were IL6, CARD9, and TNF genes with a fold of change of log_2_ > 1.

### 3.6. Seasonal Variations in the Concentration of PM in the Air of Krakow Influences the Gene Expression Profile in PBMCs of Patients and HD

**Figure 5:**
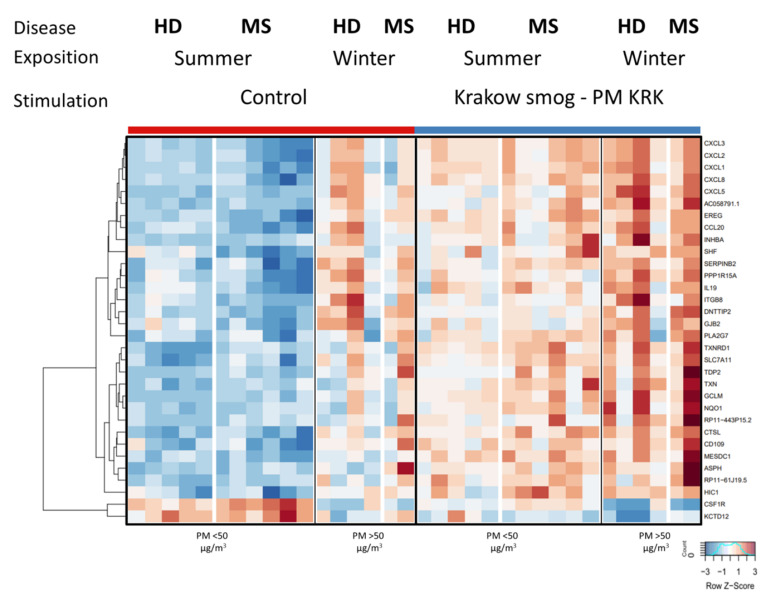
Seasonal differences in transcriptional response to PM KRK treatment between the HD and MS groups. Heatmap presents 32 genes with expression levels affected by the season. Hierarchical clustering displays differentially expressed genes in the HD and MS groups in relation to the concentration of PM in the air of Krakow in summer (50 < µg/m^3^) or winter (50 > µg/m^3^). RNAseq results are shown as a heatmap and include transcripts with a genome-wide significance from three-way ANOVA for the season factor (*p* < 0.05) computed on a list of 60 genes from Figure 3. The intensity of the coloured rectangles represents transcript abundance levels. The presented level is proportional to the row z-score values (between −3 and 3) as displayed on the bar below the heatmap image. The results of three-way ANOVA are presented in Table S3.

Since the air in Krakow differs in the concentration of PM in winter (daily concentration of PM > 50 µg/m^3^) and the summertime (daily concentration of PM < 50 µg/m^3^), in the next step we analyzed how the exposure of PBMCs to PM KRK affects the gene expression profile in relation to the high vs. low air pollution season when the blood was collected (Figure 5). This analysis was performed only for MS patients, where the blood from six of them was collected in the summer and from two others in the wintertime. In the case of HD, five persons were enrolled in the summer and four in the wintertime. Transformed FPKM values for each analyzed gene, results of ANOVA, and post-hoc analyses have been presented in Table S3.

The data show that the gene expression profile of control PBMCs (not exposed to PM KRK) in HD and MS groups differed between the seasons. The transcripts’ abundance levels are generally higher in the wintertime (Figure 5). Interestingly, the seasonal effect was then enhanced by in vitro stimulation of the cells with PM KRK. The interaction of both the factors (season and stimulation) was significant for a large part of these genes (24 out of 60 genes, nominal *p*-value < 0.05). Therefore, it seems that in vitro stimulation with PM KRK has a similar effect on the cells regardless of the season of blood collection (winter and summer); however, the basal levels of expression varied between the seasons. Moreover, the identified genes whose expression was affected by stimulation according to the concentration of PM in the air of Krakow are involved in specific intracellular pathways, including regulation of extracellular matrix by Interleukin-1 (e.g., C3 and CCL7) and transcripts related to prion diseases (e.g., EGR1 and NOTCH1).

## 4. Discussion

The specific geographical location of Krakow, the biggest city in Lesser Poland, promotes the accumulation of air pollutants, especially those from solid fuel combustion, automotive, or local industries. The air pollutions that are inhaled impose detrimental effects on the health and life quality of the local population, taking part in the initiation, development, and exacerbation of many pathologies, including allergies, and cardiovascular and autoimmune disorders [1,2,3]. So far, our studies have documented a strong polarization of CD4+ T cells into Th1 and Th17 subsets of HD after exposure to standard PM materials, differing in the content of the organic components [29]. More recently, we assessed the effect of local air pollution in Krakow on the activity of CD4+ T cell subsets in patients with AS, RA, and MS and confirmed the previous observation also in these groups of patients [30]. Moreover, we have shown that polarization of CD4+ T cells in response to PM requires monocytes and their accessory functions [29,30]. These data and further evidence pointing to the role of the urban areas’ pollution in the prevalence of civilization diseases [45,46,47,48,49,50,51], prompted us to investigate the impact of Krakow smog (PM KRK) on the gene expression profile of PBMCs from patients with AS, RA, and MS. This was determined by RNA sequencing. The analysis revealed a robust regulation of the gene expression profile in PBMCs after their treatment with PM KRK, which was detected both in patients and HD. Similar changes were observed in the study on endothelial cells after their exposure to PM 2.5 [52]. In this context, Huang et al. documented that bronchial epithelial cells undergo genome-wide alterations in gene expression and DNA methylation patterns after exposition to PM 2.5 [53]. In keeping, in atherosclerosis, the exposure to PM 2.5 was shown to affect gene expression and DNA methylation in monocytes [54]. There are also studies showing the differences in transcriptional effects between exposure to PM 2.5 and PM 10. For example, Vrijens et al. showed the alterations in gene expression and activated pathways by analyzing whole blood RNA after the volunteers’ exposure to PM 2.5 and PM 10. Nonetheless, the study was performed on healthy donors only [55]. Another study showed that the transcriptome profile of human bronchial epithelial BEAS-2B cells exposed to PM differs between winter and summer; however, the analysis compared the effect of PM 2.5 in winter with PM 10 in summer [56]. 

In our study, a multidirectional analysis of the gene expression profile with respect to the disease and stimulation factors as well as their interaction, was performed. In all these comparisons, we detected a set of genes differentially expressed between the groups, both in control, under non-stimulating conditions, and after in vitro stimulation with PM KRK. These genes are involved in multiple biological and molecular signaling pathways, including chemokine and cytokine signaling, cytokine-cytokine receptor interaction, cell adhesion, regulation of apoptosis, Toll-like receptor signaling, cell metabolism, and Th1 and Th2 cell differentiation.

Based on the previous results concerning the effect of Krakow air pollution on proinflammatory cytokine production [30], here we performed an analysis of the group of transcripts encoding factors involved in this process. Our results revealed that PBMCs stimulated with PM KRK showed increased expression of HIF1A, TICAM1, IL6, and TNF in all studied disease groups. The most pronounced effect was observed for IL6 and TNF in AS and MS patients. These results correlate with our previous observation showing the increased TNF-α and IL6 release after PBMCs exposure to PM KRK [30]. PM-induced release of proinflammatory cytokines, including TNF-α, IL-1, IL-6, and IL-8 was also documented by others [57,58] and the increased secretion of TNF-α, IL-6, and IL-8 by macrophages, lymphocytes, natural killer cells, and vascular smooth muscle cells was involved in the pathogenesis of atherosclerosis [59,60]. Furthermore, Hu et al. observed that PM 2.5 induced proinflammatory cytokine production in mice, which correlated with the initial step of AS [61]. In the case of MS, TNF-α is one of the mediators of the inflammatory response and is important in the pathogenesis and progression of this disease [62]. Additionally, TNF-α and IL-6 are also the main pathogenic cytokines in RA, having a destructive effect on bones [63]. HIF1A expression has been shown to correlate with inflammation in RA and AS [64,65]. Apart from upregulated genes, we also observed reduced expression of several genes involved in positive regulation of inflammation in all groups, including TLR6 and IL17RA. Overall, our analysis shows that genes regulated by PM KRK stimulation are enriched with those controlling the cytokine production involved in inflammation. Moreover, the RA and AS patients’ cells show higher induction of this response by PM KRK than MS patients and HD.

Our results also indicate the crucial role of seasonal variation of the PM concentration in the air of Krakow on the gene expression profile in MS patients as well as HD. However, due to the small number of subjects in particular groups, obtained results should be treated with caution. Nonetheless, in vitro stimulation with PM KRK seems to enhance the basic level of gene expression profile, which is higher in winter than in summer. These results indirectly corroborate the data showing a higher concentration of air pollution in the wintertime is associated with the increased manifestation of MS [28,66]. 

In conclusion, our results indicate significant differences in profiles of gene expression between the analyzed groups of patients and healthy controls. Additionally, in vitro treatment of human PBMCs with PM KRK clearly affects the transcriptome profile in the studied groups. PM KRK seems to upregulate the transcription of factors involved in the pro-inflammatory response and genes related to the regulation of cell metabolism and differentiation. Interestingly, the observed alterations in gene expression seem to be dependent on the seasonal concentration of PM in the air of Krakow. The regulated genes indicate biological mechanisms potentially involved in the development and progression of inflammatory and autoimmune disorders in the residents of areas with high air pollution.

## Figures and Tables

**Table 1:** Biological pathways enriched in genes (based on BioPlanet resource) with differential response of PBMCs to PM KRK treatment. The list of genes for the enrichment analysis included all genes with FDR corrected *p* < 0.05 from two-way ANOVA for the interaction (the list contained 131 genes, including genes presented in Figure 4). The top pathways (adjusted *p* < 0.1) are presented in this table. Full results are presented in Table S2.

Pathway	Genes	Overlap	Adjusted *p* Value
Interleukin-2 signaling pathway	IL10, CARD9, IL24, INPPL1, GZMB, PDE4DIP, CYTH4, PSAT1, NFIC, UCP2, FYN, TLR6, GPR18, ADA	14/847	0.07
T cell receptor regulation of apoptosis	IL10, MSR1, EGR1, IL1A, ST14, IL23A, DDAH2, CARD9, GZMB, TLR6, IER2, ADA	12/603	0.04
Cytokine-cytokine receptor interaction	IL10, IL1A, TNFSF14, CCL7, IL23A, IL24, IL19, MET	8/265	0.03
Interleukin-1 regulation of extracellular matrix	C3, IL1A, SERPINB2, CCL7, PTX3, RHOB	6/120	0.03
Interleukin-5 regulation of apoptosis	C3, EGR1, IL1A, SDC4, TLR6, IER2	6/144	0.03
Interleukin-23-mediated signaling events	IL23A, IL24, IL19	3/37	0.09

**Table 2:** The log_2_ fold change of 12 genes regulated by stimulation (FDR *p* < 0.01) associated with the positive regulation of cytokine production involved in inflammatory response (GO:1900017). The treatment-induced alterations in transcripts abundance levels in each disease group are presented.

Gene Name	HD	AS	RA	MS
HIF1A	0.38 *	0.55 *	0.24	0.49
TICAM1	0.22	0.46 *	0.24	0.49
IL6	2.88 *	4.3 *	3.22 *	4.26 *
NOD2	−0.64 *	−0.72 *	−0.66	−0.37
STAT3	−0.04	0.24	0.36	0.35
CLEC7A	0.04	−0.49 *	−0.9 *	−0.53
MYD88	−0.42 *	−0.21	−0.26	−0.16
TLR6	−0.12	−0.96 *	−0.53 *	−0.71 *
IL17RA	−0.29 *	−0.47 *	−0.28	−0.35
CARD9	−0.23	−1.7 *	−0.79	−0.85
GPSM3	−0.04	−0.26 *	−0.25	−0.42 *
TNF	0.97 *	1.84 *	1.16	1.66 *

* Significant in post-hoc analysis.

## Supplementary Materials

The following supporting information can be downloaded at: https://www.mdpi.com/article/10.3390/cells11162586/s1, Table S1: The results of gene expression profiling. Transformed FPKM values for each of the analyzed genes for all the analyzed subjects: healthy donors (HD), patients with atherosclerosis (AS), rheumatoid arthritis (RA), and multiple sclerosis (MS). The results of ANOVA for disease and stimulation factors, interaction, as well as post-hoc test results are included. Table S2: The list of enriched biological pathways in groups of genes altered by disease and stimulation factors and their interaction. The functional enrichment analyses performed with the Enrichr tool for the differentially expressed genes (are presented in Figure 2 and Figure 3). The table consists of enriched terms (based on BioPlanet 2019 dataset), the number of input genes in the pathway (overlap), the *p* value, the adjusted *p*-value, odds ratio, the combined score (computed as logarithm from *p*-value from the Fisher’s exact test multiplied by the z score of the deviation from the expected rank), and the names of overrepresented genes. The terms with adjusted *p* < 0.1 were considered enriched. Table S3: Results of three-way ANOVA test for each of the 60 analyzed genes for HD and MS subjects with an additional season factor (the exposure of PBMCs to Krakow air pollution in seasons with the concentration of particulate matter (PM) lower than the daily limit (PM < 50 µg/m^3^) and in seasons with the higher concentration than 50 µg/m^3^).
